# Current Advances in the Diagnosis and Treatment of Nonerosive Reflux Disease

**DOI:** 10.1155/2013/653989

**Published:** 2013-07-11

**Authors:** Chien-Lin Chen, Ping-I Hsu

**Affiliations:** ^1^Department of Medicine, Buddhist Tzu Chi General Hospital and Tzu Chi University, Hualien 970, Taiwan; ^2^Division of Gastroenterology, Department of Internal Medicine, Kaohsiung Veterans General Hospital and National Yang-Ming University, Kaohsiung 813, Taiwan

## Abstract

Nonerosive reflux disease (NERD) is a distinct pattern of gastroesophageal reflux disease (GERD). It is defined as a subcategory of GERD characterized by troublesome reflux-related symptoms in the absence of esophageal mucosal erosions/breaks at conventional endoscopy. In clinical practice, patients with reflux symptoms and negative endoscopic findings are markedly heterogeneous. The potential explanations for the symptom generation in NERD include microscopic inflammation, visceral hypersensitivity (stress and sleep), and sustained esophageal contractions. The use of 24-hour esophageal impedance and pH monitoring gives further insight into reflux characteristics and symptom association relevant to NERD. The treatment choice of NERD still relies on acid-suppression therapy. Initially, patients can be treated by a proton pump inhibitor (PPI; standard dose, once daily) for 2–4 weeks. If initial treatment fails to elicit adequate symptom control, increasing the PPI dose (standard dose PPI twice daily) is recommended. In patients with poor response to appropriate PPI treatment, 24-hour esophageal impedance and pH monitoring is indicated to differentiate acid-reflux-related NERD, weakly acid-reflux-related NERD (hypersensitive esophagus), nonacid-reflux-related NERD, and functional heartburn. The response is less effective in NERD as compared with erosive esophagitis.

## 1. Definitions of Gastroesophageal Reflux Disease and Nonerosive Reflux Disease

Gastroesophageal reflux disease (GERD) has been defined in the Montreal Consensus Report as a chronic condition that develops when the reflux of gastric contents into the esophagus in significant quantities causes troublesome symptoms with or without mucosal erosions and/or relevant complications [1]. The typical symptoms of GERD are recognized as heartburn and/or acid regurgitation. GERD is a common disorder with its prevalence, as defined by at least weekly heartburn and/or acid regurgitation, estimated to range from 10 to 20% in western countries and is less than 5% in Asian countries [2]. However, it has been demonstrated that GERD is emerging as a leading digestive disorder in Asian countries [3] and has an adverse impact on health-related quality of life [4].

It is noteworthy that symptoms and esophageal lesions do not necessarily exist together. A proportion of patients with erosive esophagitis have no symptoms, whereas 50–85% of patients with typical reflux symptoms have no endoscopic evidence of erosive esophagitis [5]. The latter group of GERD patients is considered to have nonerosive reflux disease (NERD) [1]. 

 The Vevey Consensus Group defined NERD as a subcategory of GERD characterized by troublesome reflux-related symptoms in the absence of esophageal erosions/breaks at conventional endoscopy and without recent acid-suppressive therapy [6]. There are some important developments that have emerged in the field of GERD with emphasizing the importance in managing those patients with NERD. It has been observed that most of the community-based GERD patients appear to have NERD [7]. In addition, previous studies have shown that NERD patients appear to be less responsive to proton pump inhibitors (PPIs) as compared with patients with erosive esophagitis [8]. 

The axiom “no acid, no heartburn” is not theoretically proper [9, 10]. Heartburn has been demonstrated as a cortical perception of a variety of intraesophageal events [11]. Subjects with heartburn without erosive esophagitis represent a heterogeneous group of patients of whom some may not have gastroesophageal-reflux- (GER-) related disorder [12–15]. In clinical practice, patients with reflux symptoms and negative endoscopic findings can be classified as (1) acid-reflux-related NERD (increased acid reflux), (2) weakly acid-reflux-related NERD (weakly acid reflux with positive symptom association; hypersensitive esophagus), (3) nonacid-reflux-related NERD (nonacid reflux with positive symptom association), and (4) functional heartburn (no associations between symptoms and reflux) (Table 1) [13]. The Rome II committee for functional esophageal disorders defined functional heartburn as an episodic retrosternal burning in the absence of pathologic GERD, pathology-based motility disorders, or structural explanations [12]. Patients with functional heartburn should be excluded from NERD because their symptoms are not related to GER.

## 2. Natural History of NERD

Recent studies regarding natural history of NERD are limited with some shortcomings including retrospective design, irregularity in follow-up, and confounding with use of medication. Very low proportion of NERD patients (3–5%) develops erosive esophagitis with the duration up to 20 years with intermittent use of antireflux therapy [16, 17]. 

 In a recent retrospective study on 2306 GERD patients with at least two separate upper endoscopies during a mean follow-up of 7 years, it was shown that most of the patients remained unchanged, while only 11% of patients worsened [18]. Similarly, the other study on patients with mild erosive esophagitis for a mean duration of 5.5 years suggests that, even within the different gradings of erosive esophagitis, the progression to severe disease is uncommon over time [19]. Therefore, the current notion regarding natural course of NERD indicates that the progression of NERD to severe form of GERD is uncommon, and there is no evidence to develop Barrett's esophagus over time [20].

## 3. Prevalence of NERD

It is difficult to estimate the true prevalence of NERD, since it is hard to identify community subjects with symptoms without seeking medical attention. There are several community-based studies in Europe that found that about 70% of the patients met the diagnosis for NERD [21]. Other international studies on subjects in primary care centers showed that about 50% of their enrolled patients had normal upper endoscopy [22]. A US study on subjects who had their reflux symptoms controlled by antacids alone has shown that 53% of those subjects had no erosive esophagitis on upper endoscopy [23]. From the previous studies, the prevalence of NERD is therefore estimated to be between 50% and 70% of the GERD population in western countries. In Asia, NERD is reported to affect different ethnic GERD populations such as 60% to 90% of the Chinese, 65% of the Indians, and 72% of the Malay [24]. 

## 4. Pathogenesis of NERD

Recent studies have provided greater insight into the pathophysiology and symptom generation in NERD. The major concepts in the pathophysiology we review include the pattern of mucosal response to gastric contents during reflux and on mucosal factors that may affect symptom perception. 

 Both esophageal dysmotility and hiatal hernia are less common in NERD than in erosive esophagitis [25]. The pathophysiology as reduced ability to clear acid from the esophagus following reflux events in patients with erosive disease is thus uncommon in NERD patients; however, the latter group is characterized by greater esophageal sensitivity in the proximal esophagus [26]. Despite no difference in gastric acid output between NERD and esophagitis [27], NERD patients have lower acid reflux when compared with patients with erosive esophagitis and Barrett's esophagus [28]. In addition, there is considerable overlap in acid exposure times between three groups of GERD patients [29]. Proximal migration of acid and nonacidic reflux seems to play a role in the symptom generation in NERD [26]. Total acid and weakly acidic reflux are greater in erosive esophagitis and Barrett's esophagus than in NERD [30], but NERD patients are shown to be of more homogenous distribution of acid exposure throughout the esophagus with greater proximal reflux [31]. With the advantage of impedance studies, NERD patients are shown to have greater proximal extent of reflux episodes (with and without prolonged esophageal acid exposure) than in healthy controls [32]. Further studies have shown greater proximal extent of reflux events which appears to be associated with symptom perception in GERD patients refractory to acid-suppression therapy [33]. Furthermore, some of the NERD patients are more sensitive to weakly acid reflux than those with erosive esophagitis [34], supporting the explanation for poor PPI response in NERD patients.

 The potential explanations for the symptom generation in NERD include microscopic inflammation, visceral hypersensitivity (stress and sleep), and sustained esophageal contractions [35]. It has been observed that acid exposure disrupts intercellular connections in the esophageal mucosa, producing dilated intercellular spaces (DIS) and increasing esophageal permeability, allowing refluxed acid to penetrate the submucosa and reach chemosensitive nociceptors [36]. DIS has been observed in both NERD and erosive disease without a significant specificity as is also found in 30% of asymptomatic individuals [37]. DIS has been found to regress with acid suppression [38]. The development of DIS may also be potentiated by bile acids and by stress [39, 40]. Stress alone may increase esophageal permeability, provoking DIS that can be enhanced by acid exposure [40]. These observations suggest a complex relationship between stress and acid exposure in the generation of reflux symptoms. 

 Peripheral receptors are shown to be mediating esophageal hypersensitivity due to acid reflux including upregulation of acid sensing ion channels, increased expression of TRPV1 receptors (transient receptor potential vanilloid type 1) [41], and prostaglandin E-2 receptor (EP-1) [42]. Peripheral and central mechanisms have also been shown to influence processing of visceral sensitivity [43]. It has been demonstrated that acute laboratory stress increased sensitivity to intraesophageal acid perception in patients with GERD [44], suggesting that the increase in perceptual responses to acid was associated with greater emotional response to the stressor. Sleep deprivation has also been shown to induce acid-related esophageal hypersensitivity [45], although there is no difference in sleep disturbance between patients with erosive esophagitis and NERD [46].

## 5. Risk Factors

GERD has been demonstrated to be influenced by genetic factors in some of the patients. In a genetic study on monozygotic twins with GERD, a significant association was found between reflux symptoms and several lifestyle factors by controlling for genetic influences [47]. Obesity was independently associated with reflux symptoms in women, but was not evident in men [47]. Smoking and physical activity at work appear to be risk factors, whereas recreational physical activity is protective [47]. Independent associations have also been reported between reflux symptoms and anxiety, depression [48], and low socioeconomic status [49]. However, it is yet unclear whether there is a specific correlation between psychological comorbidity and esophageal mucosa injury [50]. There is a higher than expected prevalence of irritable bowel syndrome (IBS) in patients with GERD symptoms [51, 52]. A recent population-based study confirmed a significant overlap between reflux symptoms and IBS, with both occurring together more frequently than expected [53].

 It appears that it is the NERD group that contributes most to the phenomenon as it is the predominant phenotype of patients with GERD symptoms, whereas some patients with erosive esophagitis may have no symptoms. Although an earlier work has attempted to compare clinical characteristics of NERD patients with those of erosive diseases patients in the same population, the potentially confounding contribution from functional heartburn has not been fully controlled [54]. Previous studies have shown that NERD patients are more likely to be female and leaner as compared with those with erosive esophagitis [22]. NERD patients are also less likely to have a hiatus hernia and more likely to have *Helicobacter pylori* [22]. Further studies in patients with NERD and erosive esophagitis indicate that both groups of the patients appear to have distinct differences regarding clinical and physiological characteristics (Table 2) [22, 25, 55].

 Recent data from Taiwan showed higher neuroticism scores in patients with reflux symptoms (with and without esophagitis) than in patients with asymptomatic esophagitis [50]. In a further study from Hong Kong, which excluded functional heartburn, IBS was independently associated with NERD instead of erosive esophagitis [25]. In addition, NERD patients were found to have increased tendency to have functional dyspepsia, psychological disorders, and positive acid perfusion test [25]. However, clinical studies show equal influence between NERD and erosive esophagitis regarding heartburn intensity [56], quality of life [57], and sleep dysfunction [46]. 

## 6. Diagnosis of True NERD and Functional Heartburn

### 6.1. Endoscopic Image

Currently, NERD is differentiated from erosive esophagitis by white light endoscopy, and NERD is further differentiated from functional heartburn by using pH monitoring (±impedance) with symptom reflux association. Recent technological advances may improve diagnostic sensitivity regarding upper endoscopy. Due to a significant overlap in the amount of reflux episodes between patients with NERD and erosive esophagitis [30], it is suggested that mucosal changes in NERD patients may be too subtle to be detected by conventional endoscopy. A recent study has confirmed the clinical utility of magnification endoscopy with narrow band imaging (NBI) which provides detailed findings in reflux diseases which are not visible by conventional endoscopy [58]. This study has shown several subtle changes in the esophageal mucosa which were identified to be highly associated with reflux disease. NERD patients appear to have intrapapillary capillary loops and microerosions identified on NBI than controls. The notation is also evident in subgroup analysis when NERD patients and esophagitis patients were compared with controls. However, despite excellent interobserver agreement for NBI findings, the drawback of NBI alone is present as modest intraobserver agreement has been demonstrated [58]. Further studies of NBI suggest that combined NBI with conventional findings gives the resolution for improving diagnostic accuracy for NERD by upper endoscopy [59].

### 6.2. 24-Hour Impedance pH Monitoring

**Figure 1:**
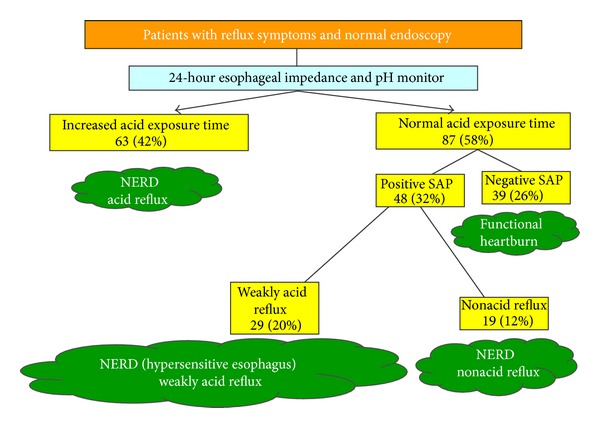
Classification of patients with reflux symptoms and normal endoscopy (SAP: symptom association probability).

 24-hour esophageal pH monitoring has been criticized for having limited sensitivity in diagnosing GERD; however, this technique is still essential for the diagnosis of NERD. The limitation of conventional pH monitoring has been overcome by combining pH with impedance monitoring [13, 60]. 24-hour impedance pH monitoring enables detection of acidic, weakly acidic, and nonacidic reflux and correlation with symptoms. This technique is able to identify three subsets of NERD (i.e., patients with an excess of acid, with a hypersensitive esophagus [to weakly acidic reflux], or with nonacid-reflux-related symptom) and patients with functional heartburn. Savarino et al. investigated the data of combined impedance pH monitoring in 150 patients with reflux symptoms and negative endoscopy under off-PPI condition (Figure 1). It was concluded that adding impedance to pH monitoring improved the diagnostic sensitivity mainly by identifying a positive symptom association probability with weakly acid or nonacid reflux in patients off PPI therapy [13]. By using this advanced technique in a group of patients with reflux symptoms not taking PPI, it was observed that the value of adding impedance measurement to standard pH monitoring could increase the observed positive symptom-reflux event association that might improve the diagnostic sensitivity of NERD [61]. From the findings previous, although combined impedance and pH measurement is necessary to reliably distinguish NERD patients from patients with functional heartburn, the test is not commonly used in general practice, and the response to PPI is more realizable than to identify those with functional heartburn [62]. Furthermore, NERD with weakly acid reflux is relatively uncommon without the condition during acid-suppression treatment.

## 7. Treatment of NERD

### 7.1. PPIs

PPIs are the most recommended and effective agents employed in the treatment of GERD. The advantage of PPIs relieving reflux symptoms is also found in NERD patients. PPIs are more effective than other acid-suppressing agents such as histamine-2 receptor antagonists (H2RAs). It has been demonstrated in NERD patients that the relative risk for PPIs versus H2RAs was 0.74 (95% CI: 0.53–1.03) for controlling heartburn [63]. 

 Initially, patients can be treated by a proton pump inhibitor (PPI; standard dose, once daily) for 2–4 weeks. If initial treatment fails to elicit adequate symptom control, increasing the PPI dose (standard dose PPI twice daily) is recommended. In patients with poor response to appropriate PPI treatment, esophageal pH (±impedance) monitoring is indicated to differentiate pathological acid reflux, acid-sensitive (hypersensitive) esophagus, and functional heartburn. The beneficial effects of PPIs in achieving symptom relief in NERD have been well documented in several studies. The rates of the relief of symptoms are shown to be 40–60% for omeprazole and rabeprazole 20 mg/day and about 30% for omeprazole 10 mg/day for 4 weeks [7, 64, 65]. By using the wireless Bravo pH monitoring, normalization of esophageal acid exposure is found in NERD patients within 48 hours after starting PPIs [66].

 NERD patients have been shown to be less responsive to PPIs as compared with patients with erosive esophagitis by approximately 20–30% after 4 weeks of the treatment [8]. The overall PPI symptomatic response rate was 36.7% (95% CI: 34.1–39.3) in NERD and 55.5% (95% CI: 51.5–59.5) in erosive esophagitis, whereas the rate of therapeutic gain was 27.5% in NERD and 48.9% in erosive esophagitis [8]. In NERD patients, the response rate appears to positively correlate with the extent of distal esophageal acid exposure with the higher symptom resolution in patients with greater acid exposure [7]. Furthermore, patients with NERD demonstrate similar symptomatic response to half and full standard dose of PPI as a prior study has shown a similar median time to first symptom relief (2 days) and to sustained symptom relief (10–13 days) for pantoprazole (20 mg/day) and esomeprazole (20 mg/day) [67]. In a subsequent study, administration of a lower dose of rabeprazole (5 mg/day) is not superior to half dose rabeprazole (10 mg/day) for heartburn relief [68]. 

 Studies have demonstrated that on-demand or intermittent PPI therapy is also an effective strategy in NERD treatment [69]. Due to the fact that most of the NERD is less likely to be progressive [20, 70], treatment for those patients can be tailored by the presence of their symptoms. Therefore, on-demand or intermittent therapy is widely used as alternative PPI treatment for NERD patients [71, 72], which also has the advantage of convenience, stable acid control, cost effectiveness, and reducing the chance of acid rebound. 

 Dexlansoprazole MR is an R-enantiomer of lansoprazole with dual delayed-release benefit in prolonging plasma concentration and pharmacodynamic effects better than those of single-release PPIs with its administration allowed at any time of the day without regard to meals. In patients with NERD, dexlansoprazole MR 30 mg daily has been shown to be more efficacious than placebo in controlling heartburn [73].

### 7.2. Novel Therapeutic Modalities

 There are novel therapeutic modalities developed specifically for NERD patients. The targets for novel therapy are thought to be improving the competence of LES function such as new GABA-B agonists, better acid-suppression therapy, normalizing esophageal sensitivity, and augmenting esophageal motility. In patients with failure to respond to PPI treatment, it has been suggested that pain modulators like tricyclics and selective serotonin reuptake inhibitors are an alternative treatment option for controlling refractory symptoms such as heartburn and chest pain [74, 75]. However, there is no sufficient evidence to support their efficacy in PPI-failure patients. In patients with PPI failure, the use of pain modulators alone or combined with PPIs can be a treatment strategy, but further studies need to confirm such approach in PPI-failure patients. 

The role of antireflux surgery NERD has not beenwell established. In general, NERD patients are less responsive to antireflux surgery [76]. In one earlier study comparing the clinical outcome of antireflux surgery between patients with erosive esophagitis and NERD, it was demonstrated that 91% versus 56% reported heartburn resolution, 24% versus 50% reported dysphagia after surgery, and 94% versus 79% were satisfied with surgery, respectively [76].

## 8. Conclusions

The definition of GERD is well established and simply understood, whereas the NERD has been intangibly defined with more conditions needed, largely because of the increased recognition of functional heartburn due to the evolution of the Rome criteria for functional gastrointestinal disorders. NERD is generally accepted as an entity within the broader definition of GERD by excluding functional heartburn. NERD has been increasingly recognized as the most common cause of reflux symptoms in community population with impact on quality of life. Mechanisms of the symptom generation in NERD remain complex, and stress may play a role in the symptom generation. Treatment with PPIs remains the choice of the therapy in NERD patients, but may be less effective when compared with those with erosive esophagitis. The role of anti-reflux surgery in NERD remains to be further investigated and defined. PPIs therapy with intermittent or on-demand fashion can be an alternative treatment strategy in most of the NERD patients due to the relatively low risk for the progression to erosive esophagitis or Barrett's esophagus.

## Figures and Tables

**Table 1:** Classification of patients with reflux symptoms.

Classification	Distal esophageal acid exposure	Symptom correlation	Symptom response to PPI
Erosive esophagitis	Increased	(+)	Good
Barrett's esophagus	Increased	(+)	Good
NERD			
Acid reflux related	Increased	(+)	Good
Weakly acid related	Not increased	(+)	Moderate*
Nonacid related	Not increased	(+)	Poor*
Functional heartburn	Not increased	(−)	Poor

*Not well investigated.

**Table 2:** Clinical and physiological characteristics between patients with NERD and erosive esophagitis.

Characteristics	NERD	Erosive esophagitis
Gender	Female	No difference
Age (yr)	40–50	50–60
Smoking (%)	15–23	10–23
Alcohol (%)	8–59	6–64
Symptom duration (yr)	1–5	1–5
Hiatal hernia (%)	20–29	39–56
*Helicobacter pylori* (+) (%)	34–41	20–26
Resting LES pressure	Normal	Normal to low
Abnormal esophageal motility	Mild	Moderate to severe
Esophageal acid clearance	Normal	Abnormal
Distal esophageal pH (<4) (% of time)	Slightly increased	Moderately increased

NERD: nonerosive reflux disease; mild: ineffective esophageal motility alone; moderate to severe: ineffective esophageal motility and impaired bolus clearance.
